# Installing a Ketocarotenoid Branch in *Phaeodactylum tricornutum* via Functional Activation of *Chlamydomonas reinhardtii* β-Carotene Ketolase

**DOI:** 10.3390/md23120470

**Published:** 2025-12-08

**Authors:** Hengshen Chao, Rasool Kamal, Yan Wu, Danqiong Huang, Chaogang Wang

**Affiliations:** 1 Guangdong Technology Research Center for Marine Algal Bioengineering, College of Life Sciences and Oceanography, Shenzhen University, Shenzhen 518055, China; chaohengshen2023@email.szu.edu.cn (H.C.);; 2 Shenzhen Engineering Laboratory for Marine Algal Biological Development and Application, College of Life Sciences and Oceanography, Shenzhen University, Shenzhen 518060, China; 3 College of Physics and Optoelectronic Engineering, Shenzhen University, Shenzhen 518060, China; 4 Instrumental Analysis Center of Shenzhen University, Shenzhen 518060, China

**Keywords:** astaxanthin, *Phaeodactylum tricornutum*, heterologous expression, ketocarotenoids, plastid targeting, CrBKT

## Abstract

Astaxanthin is a high-value ketocarotenoid antioxidant, but its industrial production from *Haematococcus pluvialis* is constrained by multi-stage cultivation and a rigid cell wall that hinders downstream extraction. The marine diatom *Phaeodactylum tricornutum*, which lacks these limitations, represents a promising alternative chassis because it grows fast, lacks a recalcitrant wall, and supports efficient pigment accumulation. This study establishes a functional ketocarotenoid biosynthetic branch in *P. tricornutum* through rational metabolic engineering. To address challenges in protein targeting posed by the host’s complex plastid architecture, we performed heterologous expression of the *Chlamydomonas reinhardtii* β-carotene ketolase (CrBKT), fused at its N-terminus to bipartite transit peptides derived from two endogenous proteins. Western blotting and UPLC-MS/MS analysis confirmed that only the transit peptide fused constructs produced stable protein and functional activity, whereas the native CrBKT failed. The rationally engineered strain successfully accumulated ~45 µg/g DCW of canthaxanthin and ~15 µg/g DCW of astaxanthin. Metabolomic profiling revealed a 50% reduction in fucoxanthin, indicating a substantial redirection of metabolic flux from the native pathway toward the engineered ketocarotenoid branch. This work establishes *P. tricornutum* as a viable platform for ketocarotenoid production and highlights the critical role of evolution-aware plastid targeting in heterologous pathway reconstruction within complex algal systems.

## 1. Introduction

Astaxanthin is a structurally unique ketocarotenoid with outstanding antioxidant properties and broad bioactivities [1]. These include anti-inflammatory, cardioprotective, and immunomodulatory effects [2]. These attributes have driven its growing demand in nutraceuticals, pharmaceuticals, and aquaculture [1]. Compared to chemically synthesized counterparts, natural astaxanthin exhibits superior bioavailability and regulatory acceptance. Chemically synthesized astaxanthin is typically a mixture of stereoisomers (3S,3′S, 3R,3′R, and meso), whereas microalgal astaxanthin consists primarily of the 3S,3′S isomer, which possesses higher antioxidant activity [1]. Furthermore, natural astaxanthin accumulates as fatty acid esters, which enhances its stability and absorption in the human body, unlike the free form found in synthetic products, conferring higher market value and consumer preference [1]. However, the limited natural supply and high production costs of astaxanthin have spurred increasing interest in developing metabolically engineered systems for its sustainable biosynthesis [3].

Currently, nearly all commercial natural astaxanthin is derived from the freshwater green alga *H. pluvialis* [3]. Although this microalga can accumulate astaxanthin under stress conditions, its production process suffers from critical bottlenecks. The two-stage cultivation—comprising a slow “green phase” followed by stress-induced accumulation—results in a lengthy cycle, high sensitivity to environmental fluctuations, and poor yield stability [4,5]. More critically, during stress, *H. pluvialis* develops a highly resistant cell wall that significantly hinders product extraction [6], making downstream processing energy-intensive and costly. These challenges severely limit the economic scalability of *H. pluvialis*-based astaxanthin production.

An emerging solution lies in developing alternative microalgal platforms with improved growth and processing characteristics [7]. The marine diatom *P. tricornutum* has attracted attention as a next-generation synthetic biology chassis, owing to its fast growth, well-established genetic toolbox, and transparent metabolic pathways [8]. It has been successfully engineered to produce various high-value compounds, such as triterpenes, PUFAs, cannabinoid, therapeutic proteins and antimicrobial peptides demonstrating its metabolic plasticity [9]. Moreover, *P. tricornutum* lacks the highly recalcitrant cell wall found in *H. pluvialis*, facilitating more accessible extraction [10]. The diatom also supports target compound accumulation under single-stage [11], non-stressed cultivation in f/2 medium, thereby eliminating the need for environmental stress induction. This streamlined process holds promise for reducing production costs while improving yield, positioning *P. tricornutum* as a favorable platform for natural astaxanthin biosynthesis.

*P. tricornutum* possesses a native carotenoid biosynthesis pathway centered on the β-carotene branch, leading to the production of its major light-harvesting pigment, fucoxanthin [12]. Metabolic engineering efforts in this organism have established multi-layered strategies, including precursor enhancement (e.g., overexpression of PSY) [13], downstream flux modulation (e.g., VDE, VDR) [14], and targeted gene editing (e.g., ZEP2, ZEP3) to redirect xanthophyll flux [15]. While these studies have significantly improved pigment content and pathway flexibility, there have been no successful attempts to reconstruct high-value ketocarotenoid branches, particularly astaxanthin, in this host.

Analysis of the metabolic pathway shows that it synthesizes its primary pigment, fucoxanthin, via the β-carotene branch [12]. Consequently, the key precursors for astaxanthin synthesis, β-carotene and its downstream product zeaxanthin, are already abundantly present as metabolic intermediates within the plastid stroma. However, genomic analysis confirms the pathway lacks the key enzyme required for the final step: β-carotene ketolase (BKT) [16].

**Figure 1:**
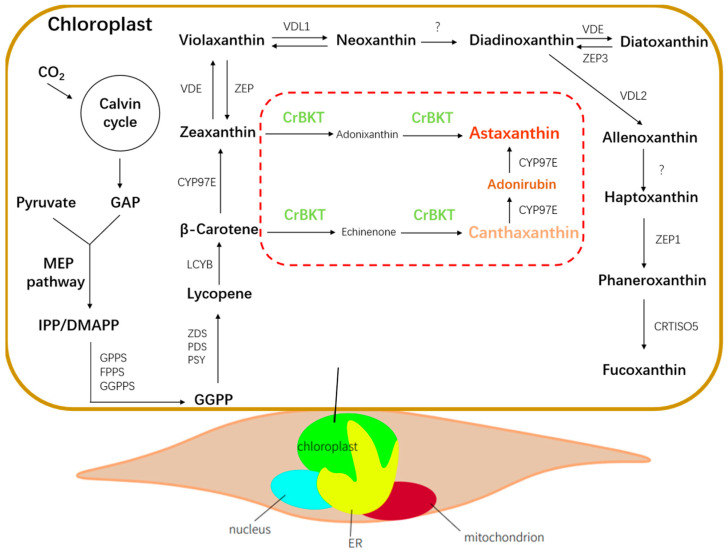
Schematic representation of the carotenoid biosynthetic pathway in *P*. *tricornutum* was drawn based on recent studies on fucoxanthin biosynthesis in diatoms [12] and the engineered ketocarotenoid branch introduced in this study. Endogenous enzymes are shown in black; the heterologous *CrBKT* introduced into the plastid is shown in green.

The CrBKT, a membrane-bound enzyme from *C. reinhardtii,* was previously shown to exhibit high catalytic efficiency in ketolating zeaxanthin, with reported conversion rates exceeding 85% in heterologous systems such as *Arabidopsis* and *Physcomitrella* [17,18]. Functional expression of CrBKT in *P. tricornutum* represents a critical step toward building a novel ketocarotenoid pathway in this host (Figure 1).

However, heterologous expression of membrane-associated carotenoid enzymes is often limited by subcellular targeting and folding constraints [19]. Astaxanthin biosynthesis occurs in the plastid stroma, and *P. tricornutum* features a secondary endosymbiotic plastid enclosed by four membranes [20]. Nuclear-encoded proteins require a bipartite targeting sequence (BTS), a signal peptide (SP) followed by a transit peptide (TP) to reach the correct subplastidial compartment [21]. In contrast, CrBKT from *C. reinhardtii* carries a native transit peptide suited for a simpler two-membrane chloroplast [22] and is unlikely to be fully compatible with the host import machinery. We therefore hypothesized that fusing CrBKT to a functional *P. tricornutum*-derived BTS would enable proper plastid targeting and enzymatic function.

In this study, we validated this hypothesis using comparative constructs with and without N-terminal plastid-targeting sequences fused to CrBKT and evaluated their performance. Functional expression and correct localization enabled the first successful de novo biosynthesis of astaxanthin and canthaxanthin in *P. tricornutum*. Multi-omics analyses (genomic, transcriptomic, proteomic, and metabolomic) confirmed that plastid localization is a prerequisite for establishing a functional ketocarotenoid branch. Furthermore, metabolomic profiling revealed a marked redirection of metabolic flux from the native fucoxanthin pathway toward the engineered ketocarotenoid branch. This work not only expands the carotenoid biosynthetic potential of *P. tricornutum* but also establishes it as a promising marine chassis for cost-effective astaxanthin production. Importantly, this study provides a practical demonstration of effective plastid targeting in a complex secondary endosymbiont, offering valuable insights for future heterologous pathway design in similar systems.

## 2. Results

### 2.1. Rational Construct Design and Multi-Level Molecular Validation of Functional CrBKT

**Figure 2:**
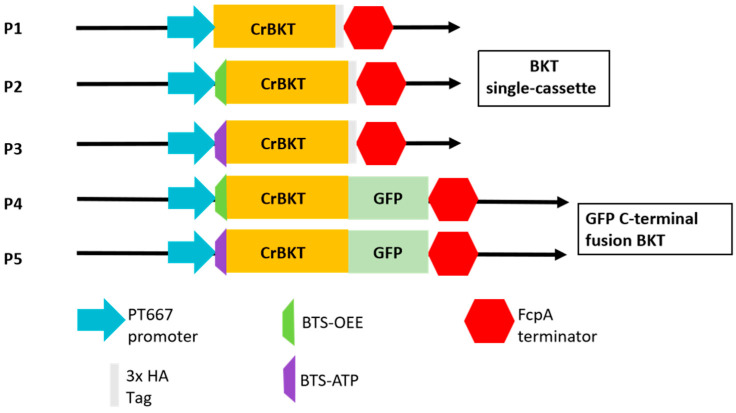
Schematic diagram of the expression vectors. All constructs were designed for stable genomic integration. P1: Negative control expressing CrBKT with its native *C. reinhardtii* transit peptide. P2/P3: Experimental constructs fusing CrBKT to endogenous *P. tricornutum* bipartite transit sequences (BTS-OEE and BTS-ATP, respectively). P4/P5: C-terminal GFP fusion constructs, testing translational fusion with BTS-OEE/BTS-ATP. All *CrBKT* cassettes (P1–P5) were driven by the pt667 promoter and (P1–P3) C-terminally fused with a 3x HA tag for detection. All cassettes utilized the FcpA terminator.

To install a heterologous ketocarotenoid biosynthetic branch in *P. tricornutum*, we designed a series of expression vectors to test the functional expression of the β-carotene ketolase (CrBKT) from the primary endosymbiont *C. reinhardtii* (Figure 2). It was hypothesized that the *C. reinhardtii* native transit peptide would be incompatible with the four-membrane plastid import machinery of the secondary endosymbiont *P. tricornutum*. To test this, a negative control vector (P1) expressing CrBKT with its native transit peptide was constructed. Concurrently, the primary experimental constructs (P2, P3) were designed by fusing the codon-optimized *CrBKT* gene to two different *P. tricornutum* endogenous bipartite transit sequences BTS-OEE and BTS-ATP (the oxygen-evolving enhancer protein and the ATP synthase γ-subunit) [21,23], which are known to target proteins to the plastid stroma. To investigate subcellular localization and protein stability, we also generated C-terminal GFP fusion constructs (P4, P5). All the five constructs (P1–P5) were driven by the strong, constitutive pt667 promoter [24].

**Figure 3:**
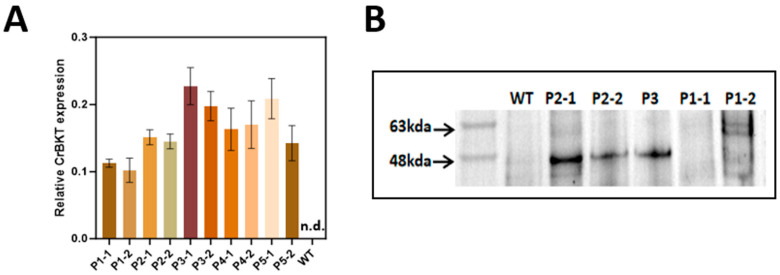
CrBKT transcript levels in wild-type and selected *P. tricornutum* transformants. For each plasmid construct (P1–P5), two top-expressing CrBKT transformants (lines “1” and “2”) were selected based on the qRT-PCR screening shown in Supplementary Figure S1. Relative CrBKT expression was quantified by qRT-PCR using the TBS gene as an internal reference, and calculated as 2^−ΔCt^ (ΔCt = Ct_CrBKT − Ct_TBS). Bars represent mean ± SD (*n* = 3 biological replicates, each measured in technical triplicate). CrBKT transcripts were not detected in the wild-type (WT) strain (n.d., Ct > 40), which is therefore shown at zero for visualization. (**B**) Analysis of CrBKT protein expression and stability. Total protein was extracted from WT and representative transformants (P1–P5) and analyzed by immunoblotting using an anti-HA/GFP antibody. The theoretical size of the precursor CrBKT-HA including the diatom BTS is ~56 kDa; upon plastid import the BTS is processed, yielding a mature form of ~50 kDa. Clear bands corresponding to CrBKT were observed only in lines P2, P3. No detectable protein was found in P1, P4, or P5, despite high transcript levels in (**A**). Full, unprocessed blot images are provided in Supplementary Figure S4.

These constructs were introduced into wild-type *P. tricornutum* via biolistic transformation. For each construct (P1–P5), approximately 200 colonies were initially screened, from which ~30 transformants per construct (~10–15% efficiency) were confirmed positive for genomic integration of *CrBKT* by PCR (Supplementary Figure S1). Transcriptional activation was subsequently quantified by RT-qPCR (Figure 3A). Based on qPCR results, the 1–2 lines with the highest *CrBKT* transcript levels from each construct group were selected for further protein-level validation via Western blot. All constructs including the native transit peptide control (P1), the GFP fusion constructs (P4, P5), and the plastid-targeted experimental lines (P2, P3) showed robust *CrBKT* mRNA accumulation.

Despite this, Western blot analysis revealed divergent protein expression across constructs. Anti-HA immunoblotting detected strong CrBKT protein bands in the plastid-targeted lines (P2, P3), but not in P1 (native transit peptide) (Figure 3B). The GFP-fusion constructs (P4, P5), which lacked HA tags, were analyzed separately by anti-GFP blotting (Supplementary Figure S3). No detectable fusion protein was observed in either line.

These results reveal two possible limitations in heterologous CrBKT expression. First, the native transit peptide from *C. reinhardtii* appears insufficient to support efficient plastid import in *P. tricornutum*. As a result, the non-targeted CrBKT protein likely accumulated in non-native subcellular compartments, such as the cytosol or endoplasmic reticulum, where it may have misfolded or failed to integrate into membranes. Given its hydrophobic nature as a membrane-associated ketolase [25], such mislocalization would render the protein unstable and susceptible to proteolytic degradation, thereby explaining the absence of detectable bands in the anti-HA immunoblot.

Second, the fusion of CrBKT to a large C-terminal GFP tag also abolished detectable protein expression. This may reflect structural interference that disrupted proper folding, membrane association, or translocation, which is consistent with prior reports on the sensitivity of carotenoid pathway enzymes to steric hindrance from fusion partners [26].

Because no functional protein was detected in P4 and P5, these lines were excluded from downstream metabolite analysis. In contrast, P1 was retained as a biological control to test whether trace mislocalized CrBKT might exhibit minimal activity, based on the Western blot results, representative high-expression lines from each construct group were selected for subsequent omics analyses and renamed accordingly: P2-1 was designated as O6, P3 as A3, and P1-2 as P1 (control).

Together, these findings demonstrate that stable protein accumulation requires fusion of CrBKT to an appropriate plastid-targeting signal (P2, P3), validating the necessity of correct subcellular targeting as a prerequisite for functional ketocarotenoid biosynthesis.

### 2.2. Quantitative Analysis of the Engineered Ketocarotenoid Pathway Reveals a Hydroxylation Bottleneck

**Figure 4:**
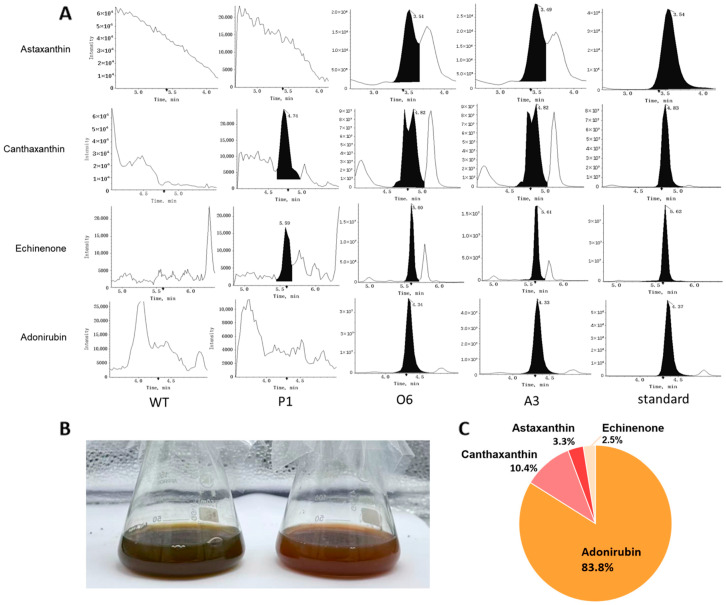
Metabolic profiling and product composition of engineered *P. tricornutum* strains. (**A**) UPLC-MS/MS chromatograms showing the detection of ketocarotenoids and their intermediates in wild-type (WT), P1 (native CrBKT transit peptide), and functional lines O6 and A3. Multiple new peaks were detected in O6 and A3 corresponding to echinenone, adonirubin, canthaxanthin, and astaxanthin, which were absent in the WT. (**B**) Representative culture images of WT (left) and a successfully engineered strain (right; representative of O6/A3) after 21 days of cultivation. Only lines with stable CrBKT protein expression (e.g., O6 and A3) developed a visible color shift from the typical brown hue of *P. tricornutum* (due to fucoxanthin) to an orange-red tone, consistent with ketocarotenoid accumulation. (**C**) Relative composition of four identified ketocarotenoids in the O6 and A3 strains. Adonirubin constituted approximately 85% of the total product pool, while canthaxanthin, astaxanthin, and echinenone represented minor fractions.

Following the molecular confirmation of functional protein expression in strains O6 and A3, we performed UPLC-MS/MS to identify and quantify the resulting metabolic products. The UPLC-MS/MS chromatograms (Figure 4A, Supplementary Figure S2) confirmed the presence of multiple new compounds in the engineered lines that were absent in the wild-type. These compounds were identified as the target ketocarotenoids canthaxanthin and astaxanthin, as well as the pathway intermediates echinenone and adonirubin. Visibly, the engineered strains also exhibited a distinct orange-red pigmentation compared to the typical brown wild-type cells (Figure 4B), consistent with the accumulation of newly synthesized carotenoids.

Absolute quantification revealed that the correctly targeted CrBKT was highly effective at pulling flux into this new pathway, generating a total ketocarotenoid pool in excess of 460 µg/g DCW in the O6 strain (Table 1). However, the product profile was unexpectedly dominated by the intermediate adonirubin, which accumulated to ~400 µg/g DCW. The other products, canthaxanthin (~45 µg/g), astaxanthin (~15 µg/g), and echinenone (~10 µg/g), were present at significantly lower levels. As visualized in a compositional pie chart (Figure 4C), adonirubin alone accounted for approximately 85% of the entire newly synthesized ketocarotenoid pool.

This product distribution provides a clear and critical insight into the pathway’s dynamics. The pathway from β-carotene to astaxanthin involves sequential ketolation (catalyzed by BKT) and hydroxylation (catalyzed by β-carotene hydroxylase, CHY). The massive accumulation of adonirubin (a di-keto, mono-hydroxy carotenoid) and canthaxanthin (a di-keto carotenoid), compared to the low titer of the final product astaxanthin (a di-keto, di-hydroxy carotenoid), strongly indicates that the hydroxylation step is the new rate-limiting bottleneck in the engineered pathway [27]. The high catalytic efficiency of the heterologous CrBKT is evident in its ability to generate over 460 µg/g of total ketolated products. However, the host’s endogenous CHY enzymes, which are evolutionarily optimized for the native fucoxanthin pathway, appear to have very low substrate affinity or catalytic efficiency for these novel, bulky ketocarotenoid substrates (i.e., converting adonirubin to astaxanthin). This identifies the co-expression of a more efficient, heterologous CHY as a primary target for future optimization, as aspect that is addressed in the Discussion section.

A particularly insightful finding emerged from the high-sensitivity analysis of the P1 negative control (native CrBKT transit peptide). As established in Section 2.1, this strain failed to produce detectable protein by Western blot (Figure 3B) or any phenotypic change (Figure 4B). However, UPLC-MS/MS analysis, which offers significantly higher sensitivity than immunoblotting, detected trace but quantifiable levels of canthaxanthin (averaging ~0.03 µg/g DCW) and its precursor echinenone (Figure 4A, Table 1). The total ketocarotenoid production in our functionally targeted strains (e.g., O6) represents a >15,000-fold increase in metabolic flux compared to the mislocalized P1 control. This result definitively confirms our central hypothesis: that correct plastid localization via an endogenous BTS is absolutely essential for high-efficiency ketocarotenoid production. The trace activity observed in P1 suggests two possibilities. The first one is that the native transit peptide is largely ineffective in the diatom, causing the majority of CrBKT to mislocalize to the cytosol or ER, where it intercepts leaking β-carotene [25]. Alternatively, a small fraction of CrBKT may have successfully entered into the plastid but failed to drive significant flux. This could be due to the low affinity of the endogenous hydroxylase (CYP97E) for the trace amounts of canthaxanthin produced. Endogenous hydroxylases can exhibit high substrate specificity for β-carotene and often possess poor catalytic efficiency towards ketolated intermediates, creating a metabolic bottleneck when substrate concentrations are low [27]. Therefore, both spatial segregation and kinetic limitations likely contribute to the stalled pathway in the P1 control.

### 2.3. Metabolomic Analysis Reveals Significant Metabolic Flux Redirection from the Native Fucoxanthin Pathway

**Figure 5:**
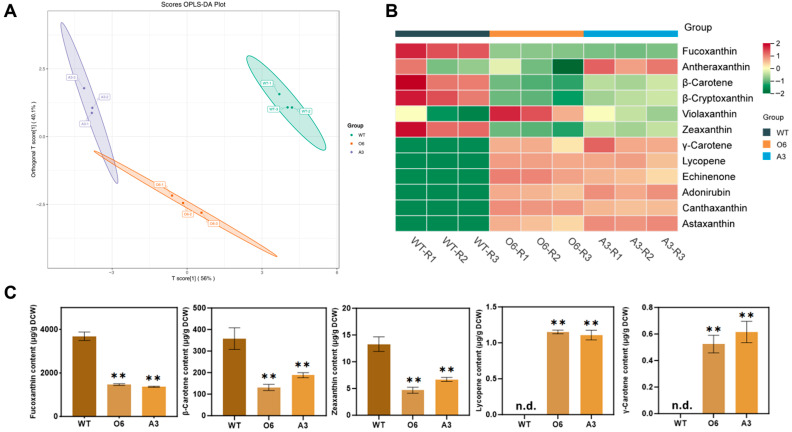
Global metabolomic and carotenoid pathway reprogramming in engineered *P. tricornutum* lines expressing CrBKT. (**A**) Orthogonal Partial Least Squares Discriminant Analysis (OPLS-DA) of global UPLC-MS/MS-derived metabolite profiles from wild-type (WT) and engineered strains (O6, A3). Each dot represents a biological replicate. (**B**) Heatmap of 12 representative carotenoids and intermediates across all samples. (**C**) Absolute quantification of key carotenoids: fucoxanthin, β-carotene, zeaxanthin, lycopene and γ-carotene. Data represent mean ± SD (*n* = 3). Statistical significance was assessed by one-way ANOVA with Tukey’s post hoc test: *p* < 0.01 (**), “n.d.” indicates not detected. Detailed quantitative values and full statistical analysis are provided in Supplementary Tables S2 and S3.

To assess the systemic impact of CrBKT expression on the host’s native carotenoid pathway, we conducted a comprehensive UPLC-MS/MS-based metabolomic analysis comparing the functionally engineered lines with the wild-type (WT). A Principal Component Analysis (PCA) of the global metabolite profiles demonstrated a clear and significant separation between the engineered strains and the WT (Figure 5A). This confirms that the functional integration of a single heterologous enzyme induced a robust and reproducible shift in the overall cellular metabolism.

To dissect the underlying flux redistribution, we profiled 12 carotenoids using a heatmap (Figure 5B), which clearly showed a reciprocal pattern: ketocarotenoids (e.g., astaxanthin, canthaxanthin, adonirubin, echinenone) were strongly upregulated in engineered strains, while native pigments particularly fucoxanthin, β-carotene, and zeaxanthin were depleted.

A more detailed quantification of five key carotenoids, fucoxanthin, β-carotene, zeaxanthin, lycopene, and γ-carotene, revealed the precise nature of this metabolic shift (Figure 5C). The powerful metabolic “pull” from the highly active CrBKT enzyme led to a ~50–60% reduction in both β-carotene (from ~358 to ~130–190 µg/g DCW) and zeaxanthin (from ~13.3 to ~5–7 µg/g DCW). These shared precursors are critical substrates not only for CrBKT, but also for the native fucoxanthin biosynthetic pathway.

This severe depletion of the precursor pool had a direct and profound consequence on the competing native pathway. As anticipated, the synthesis of the host’s primary light-harvesting pigment, fucoxanthin, was crippled. Quantitative data showed that fucoxanthin levels decreased by ~55–60% in the engineered strains (e.g., ~1400 µg/g DCW) compared to the WT (~3686 µg/g DCW). The proportional 50–60% drop in both the precursor pools β-carotene, zeaxanthin) and the final native product (fucoxanthin) provides unequivocal quantitative evidence of a massive metabolic flux redirection, confirming that the CrBKT-driven pathway successfully “hijacked” more than half of the carbon flux previously destined for fucoxanthin synthesis. Interestingly, other xanthophyll cycle intermediates, such as violaxanthin and antheraxanthin, remained stable or increased slightly, suggesting a possible compensatory stress response by the cell to the loss of its main photopigment.

Furthermore, this intense downstream pull exposed a previously hidden bottleneck in the upstream biosynthetic pathway. In wild-type *P. tricornutum*, the acyclic intermediates lycopene and γ-carotene are typically present at extremely low or undetectable levels under standard growth conditions [28,29]. However, in the engineered strains, these intermediates accumulated to quantifiable levels (~1.1 µg/g DCW and ~0.6 µg/g DCW, respectively). Given that the total carotenoid pool in the engineered lines decreased by nearly 50% (from ~4100 µg/g DCW in WT to ~2100 µg/g DCW in O6/A3), the appearance of lycopene does not signify an increase in total upstream flux. Instead, it indicates that the rapid consumption of β-carotene (the product) placed immense pressure on the enzyme catalyzing its formation, lycopene β-cyclase (LCYB). This exposed the catalytic capacity of LCYB as a new rate-limiting step, causing its substrates (lycopene and γ-carotene) to “spill over” and accumulate for the first time.

Taken together, these results highlight the systemic consequences of introducing a high-flux heterologous pathway into *P. tricornutum*. The data suggest that LCYB (upstream) and CHY (downstream) may represent newly exposed bottlenecks for further strain optimization.

## 3. Discussion

In this study, we report the first successful biosynthesis of high-value ketocarotenoids, astaxanthin and canthaxanthin, in the marine diatom *P. tricornutum* through a rational metabolic engineering strategy. The significance of this achievement extends beyond expanding the metabolic engineering repertoire of *P. tricornutum* as an established synthetic biology chassis; it establishes a promising alternative platform for the industrial production of natural astaxanthin. Currently, commercial production is dominated by the freshwater microalga *H. pluvialis* [6], a system limited by two fundamental bioprocessing bottlenecks. First, *H. pluvialis* cultivation relies on a complex and inefficient two-stage process [30]. Second, and more critically, stress-induced astaxanthin accumulation triggers the formation of a rigid, non-hydrolyzable cell wall, which renders downstream extraction highly energy-intensive and costly [31]. The *P. tricornutum* chassis developed in this study circumvents both limitations: it allows product accumulation under single-phase, non-stressed cultivation and lacks the thick-walled encystment barrier [32], thereby offering a more streamlined and potentially cost-effective production platform.

Regarding downstream processing, the strategy depends on the target purity. For aquaculture feed, the crude lipid extract containing the ketocarotenoid mixture can be utilized directly without fractionation. However, for high-value nutraceutical applications requiring purified astaxanthin, standard industrial protocols can be adapted. This typically involves saponification to hydrolyze esters, followed by chromatographic separation to isolate astaxanthin from intermediates based on their distinct polarities [7]. Crucially, the absence of a recalcitrant cell wall in *P. tricornutum* eliminates the need for the energy-intensive disruption steps required for *H. pluvialis* cysts [31], thereby streamlining the initial extraction and making the overall purification process more cost-effective. However, achieving this was not a simple gene insertion, as revealed in our “Results”. It required a rational protein engineering strategy to overcome the evolutionary barrier between the primary endosymbiont enzyme source (*C. reinhardtii*) and the secondary endosymbiont host (*P. tricornutum*). The four-membrane plastid of *P. tricornutum* necessitates a bipartite transit sequence (BTS) for correct protein import [21], a mechanism incompatible with the CrBKT native transit peptide. Our results provided a stark validation of this hypothesis: the P1 control line (native peptide) yielded only trace (~0.03 µg/g DCW) levels of canthaxanthin, while the correctly targeted strains (e.g., O6, A3) produced a total ketocarotenoid pool of over 470 µg/g DCW, a >15,000-fold increase that unequivocally demonstrates rational protein targeting as the essential prerequisite for this pathway.

Our comprehensive metabolomic analysis provided a detailed “pressure test” of the *P. tricornutum* metabolic network under the strain of this new, high-flux pathway. The data revealed a massive metabolic flux redistribution. The powerful “pull” of the CrBKT enzyme successfully “hijacked” approximately 50–60% of the carbon flux previously destined for the native fucoxanthin pathway, evidenced by the ~60% drop in the fucoxanthin end-product and a corresponding ~50–60% depletion of the shared precursor pools, β-carotene and zeaxanthin. This intense flux re-routing simultaneously exposed three critical bottlenecks that now define the roadmap for optimization: (1) Upstream bottleneck at *LCYB*: The unexpected accumulation of lycopene and γ-carotene in our engineered lines indicates that LCYB is unable to sustain the increased flux demanded by CrBKT activity. Prior studies have shown that modulation of *LCYB* expression significantly affects the β-carotene/α-carotene ratio and hence carotenoid flux partitioning in microalgae [33]. To overcome this bottleneck, overexpression of the native *LCYB* gene or introduction of a heterologous LCYB with higher catalytic turnover may be warranted [34]. (2) Downstream bottleneck at the endogenous *CHY*, evidenced by the massive accumulation of the intermediate adonirubin (~400 µg/g DCW) [27]. Balancing the ketolase:hydroxylase ratio and co-expressing a high-activity, plastid-targeted CHY that accepts 4-keto substrates are established solutions that collapse the adonirubin “reservoir” and pull carbon to astaxanthin [35,36]. In microalgae and other photosynthetic chassis, co-expression of the β-carotene ketolase and β-carotene hydroxylase is required to avoid trapping flux at canthaxanthin/adonirubin and to pull carbon to astaxanthin [37,38]; tuning the BKT: CHY expression ratio (promoters/copies/fusions) is a decisive lever. Similar “pull” optimization has been pivotal in microalgae, where tuning BKT/CHY expression markedly boosts astaxanthin. (3) A competitive bottleneck from the native *ZEP* pathway.

This “first-generation” total ketocarotenoid yield of ~470 µg/g DCW (0.047%) is a highly encouraging proof-of-concept under phototrophic conditions. This titer is already in the same order of magnitude as foundational engineering efforts in other algae, such as *C. reinhardtii* [22], and approaches the content of some heterotrophic *Chromochloris zofingiensis* strains (e.g., ~1.44 mg/g DCW) [39], all while being achieved in a chassis with superior bioprocessing traits. The clear identification of the three bottlenecks (LCYB, CHY, ZEP) provides a sophisticated “Push-Pull-Block” strategy for future work [40]. This includes (1) “Pushing” precursor supply by overexpressing LCYB and PSY, (2) “Pulling” the final product by co-expressing a heterologous CHY to convert the ~400 µg/g DCW adonirubin “reservoir” to astaxanthin, and (3) “Blocking” competition by using CRISPR/Cas9 to knock out the competing ZEP.

Nevertheless, blocking fucoxanthin biosynthesis presents a physiological trade-off. Fucoxanthin is essential for light harvesting in diatoms [41], and its complete elimination, as observed in *ZEP1* mutants, leads to impaired photosynthesis and poor phototrophic growth [29,42]. This presents a dilemma: maximizing product yield may compromise the biomass productivity necessary for scalable cultivation.

Therefore, an ultimate industrial strategy must uncouple growth from light. *P. tricornutum* is an ideal candidate for this, as it can utilize glycerol and has been successfully engineered to grow heterotrophically on glucose via expression of hexose transporters [43,44]. A superior, next-generation strain would combine the full “Push-Pull-Block” system with this heterotrophic capability. Such a strain, grown in a dark fermenter on simple sugars, would completely circumvent the “photosynthetic conflict,” allowing 100% of the carbon flux to be channeled towards astaxanthin, thereby creating a truly competitive industrial platform.

## 4. Materials and Methods

### 4.1. Strains and Growth Conditions

*Escherichia coli* TOP10 (Yeasen Biotechnology Co., Ltd., Shenzhen, China) was used to construct and propagate recombinant plasmids. The *E. coli* cells were cultured at 37 °C and 200 rpm in liquid or solid LB medium (5 g/L of yeast extract, 10 g/L of peptones, 10 g/L of NaCl, and additional 15 g/L of agar for the solid medium) containing 100 μg/mL of ampicillin (Biosharp, Beijing, China). *P. tricornutum* was grown in f/2 liquid medium as described previously [45], under rigorous shaking 100 rpm at 22 °C with continuous illumination at 50 μmol·photons·m^−2^ s^−1^.

### 4.2. Plasmid Design and Assembly

The β-carotene ketolase gene (*CrBKT*) used in this study was derived from *C. reinhardtii* (GenBank accession: AY860820.1). The expression cassette consisted of the pt667 promoter driving a codon-optimized *CrBKT* sequence, adapted to the codon usage preference of *P. tricornutum*. An N-terminal bipartite plastid targeting peptide, derived from either the oxygen-evolving enhancer protein (OEE) or ATP synthase γ-subunit (ATP) of *P. tricornutum*, was fused in-frame upstream of the *CrBKT* coding sequence. A 3×HA tag was appended at the C-terminus, and expression was terminated by the fcp*A* terminator. The selectable marker was *shble*, conferring resistance to Zeocin, and was expressed under the fcp*B* promoter with the fcp*A* terminator. Built on the pPha-T1 backbone [46], which has been widely used for nuclear transformation in *P. tricornutum*.

All plasmid constructs were assembled using Gibson assembly, performed with the NEBuilder^®^ HiFi DNA Assembly Bundle for Large Fragments (New England Biolabs, Hitchin, UK). DNA fragments for assembly were amplified by PCR using the Platinum™ SuperFi Green PCR Master Mix (ThermoFisher, Waltham, MA, USA), following the manufacturer’s protocol.

### 4.3. Nuclear Transformation and Transformants Identification

Transformation of *P. tricornutum* was carried out according to Apt et al. [47]. Firstly, 10^8^ cells/mL were harvested and concentrated, then spread as a thin lawn on f/2 agar pre-warmed plates. Gold particles diameter 0.6 µm (Biorad, Hercules, CA, USA) were coated with 5 µg plasmid DNA using 2.5 M CaCl_2_ and 0.1 M spermidine following the manufacturer’s protocol. Bombardment was performed on a Bio-Rad Biolistic PDS-1000/He Particle Delivery System at helium pressure 1550 psi, vacuum 26.5 psi, and target distance 6 cm. After 48 h recovery in antibiotic-free medium, cells were spread onto f/2 medium (1% [*w*/*v*] agar plates) containing Zeocin 100 µg/mL. Transformants appeared after 14 days at 22 °C with continuous illumination at 50 μmol·photons·m^−2^ s^−1^.

For genomic validation, DNA was extracted from transformants using the Ultra DNA Isolation Kit (Bebebio, Zhengzhou, China). Colony PCR was conducted with construct-specific primers using 2 × M5 HiPer Taq HiFi PCR Mix (Mei5 Biotechnology, Beijing, China) under the following program: 95 °C for 3 min; 35 cycles of 95 °C for 10 s, 59 °C for 20 s, and 72 °C for 15 s; followed by a final extension at 72 °C for 5 min. PCR products were analyzed on 1% agarose gels.

Total RNA was extracted using the VAZYME Magnetic Universal Plant Total RNA Kit (VAZYME, Nanjing, China), and 1 μg of total RNA was reverse-transcribed using the 1st Strand cDNA Synthesis Kit for qPCR (gDNA digester plus) (TransScript, Beijing, China). Quantitative real-time PCR (RT-qPCR) was performed using a QuantStudio™ 6 Flex System with qPCR Master Mix (TransScript, Beijing, China). Relative gene expression was calculated using the 2^−ΔCT^ method, with *P. tricornutum* TATA box binding protein (TBP) serving as the endogenous reference gene [48]. All primer sequences used for colony PCR and RT-qPCR are listed in Supplementary Table S1.

### 4.4. Western Blot Analysis

Total protein was extracted from *P. tricornutum* cell pellets using RIPA buffer supplemented with protease inhibitors. Proteins were separated by SDS–PAGE and transferred to PVDF membranes using standard protocols. After blocking with 5% skim milk in TBST, membranes were incubated overnight at 4 °C with HRP-conjugated anti-HA tag monoclonal antibody (Wuhan Sanying Biotechnology, Wuhan, China Catalog No. HRP-81290) or HRP-conjugated anti-GFP tag monoclonal antibody (Wuhan Sanying Biotechnology, Wuhan, China Catalog No. HRP-66002), depending on the construct. After washing, signals were developed using enhanced chemiluminescence (ECL) and visualized using a chemiluminescence imaging system.

### 4.5. Chromatographic Mass Spectrometric Analysis of Carotenoids in Transgenic Algae

Algal pellets were freeze-dried and ground into a homogenized fine powder using a ball mill (30 Hz, 1 min) to ensure complete disruption and extractability. For each sample, 50 mg of powder was extracted with 0.5 mL of hexane:acetone:ethanol (1:1:1, *v*/*v*/*v*) containing 0.01% butylated hydroxytoluene (BHT) as antioxidant. After concentration, the extract was redissolved in 100 μL of methanol:methyl tert-butyl ether (1:1, *v*/*v*), filtered through a 0.22 μm organic membrane, and transferred into amber vials. Chromatographic separation was performed using a YMC C30 column (3 μm, 100 mm × 2.0 mm) on a QTRAP 6500+ LC-MS/MS system (SCIEX), operated in APCI-positive mode. The mobile phases consisted of (A) methanol/acetonitrile (1:3, *v*/*v*) with 0.01% BHT and 0.1% formic acid, and (B) methyl tert-butyl ether with 0.01% BHT. The gradient elution program was: 0–3 min, 100% A; 3–5 min, 30% A; 5–10 min, 5% A; 10–11 min, return to 100% A. Flow rate was 0.8 mL/min, column temperature 28 °C, and injection volume 2 μL. Mass spectrometric parameters and ion transitions were optimized for each carotenoid standard as described previously [49]. All UPLC-MS/MS analyses were conducted by MetWare Biotechnology Co., Ltd. (Wuhan, China).

### 4.6. Qualitative and Quantitative Analysis of Carotenoid Compounds

Carotenoid compounds were identified and quantified using a targeted metabolomics approach. Compound identification was based on retention time and mass spectrometry fragmentation patterns, matched against an in-house standard database (MWDB, Metware Biotechnology, Wuhan, China). Quantification was performed using multiple reaction monitoring (MRM) mode on a triple quadrupole mass spectrometer.

For each sample, chromatographic peaks were extracted, and the corresponding peak areas were interpolated into compound-specific standard curves to calculate concentrations (µg/mL). Final carotenoid content was calculated using the following formula:

Carotenoid content (μg/g) = c × V/1000/m

where c is the compound concentration (µg/mL) obtained from the standard curve, V is the reconstitution volume (µL), and m is the mass of the dried algal biomass (g).

### 4.7. Statistical Analysis

All metabolomic datasets were normalized using unit variance (UV) scaling prior to statistical analysis. Heatmaps were generated using the ComplexHeatmap package in R (v4.3). Metabolite differences between samples were assessed using a combination of univariate (fold change analysis, Student’s *t*-test or false discovery rate adjustment) and multivariate (e.g., PCA) statistical methods.

Metabolites with significant differences (*p* < 0.05 or FDR < 0.05) and a fold change threshold (typically FC > 2 or < 0.5) were considered differentially accumulated.

## 5. Conclusions

In this study, we report the successful establishment of a functional ketocarotenoid biosynthetic branch in the marine diatom *P. tricornutum*. This work establishes *P. tricornutum* as a viable and promising biotechnological chassis for ketocarotenoid production, one that fundamentally circumvents the critical bioprocessing limitations—namely the complex two-stage cultivation and the recalcitrant cell wall that hinders extraction—of the current industrial source, *H. pluvialis*. We demonstrated that the success of this metabolic engineering strategy was contingent upon a rational protein targeting approach. Fusing the CrBKT catalytic domain to an endogenous bipartite transit sequence was proven essential for correctly localizing the enzyme to the plastid stroma, a prerequisite for functional activation that accounts for the evolutionary divergence between primary and secondary endosymbiotic plastids. Furthermore, our quantitative metabolomic analysis confirmed the functional impact of this new branch, revealing a significant 50% redirection of metabolic flux away from the native fucoxanthin pathway. While the yields from this first-generation strain (~460 µg/g DCW) are modest, our findings establish a clear and sophisticated roadmap for future optimization. This roadmap suggests that a systems-level approach, combining “Push-Pull-Block” strategies with a metabolic shift to heterotrophy, could uncouple pigment production from phototrophic limitations, paving the way for *P. tricornutum* to become a commercially competitive platform for producing astaxanthin and other high-value marine-derived compounds.

## Figures and Tables

**Table 1:** Quantification of ketocarotenoids in wild-type (WT) and engineered *P. tricornutum* lines (P1, O6 and A3). All values are presented as mean ± SD (*n* = 3), rounded to three decimal places; unit: µg/g DCW. Detailed quantitative values are provided in Supplementary Table S2.

Compound	WT	P1	O6	A3
Echinenone	0	0.020 ± 0.003	10.831 ± 0.626	8.920 ± 0.700
Adonirubin	0	0	353.348 ± 18.118	391.852 ± 17.847
Canthaxanthin	0	0.028 ± 0.004	44.846 ± 0.731	39.027 ± 0.758
Astaxanthin	0	0	13.381 ± 1.070	16.045 ± 0.286

## Data Availability

The original data are available from the correspondent author on request.

## Supplementary Materials

The following supporting information can be downloaded at: https://www.mdpi.com/article/10.3390/md23120470/s1, Figure S1: PCR-based confirmation of *CrBKT* gene integration in transgenic *P. tricornutum* lines; Figure S2: qRT-PCR–based screening of *P. tricornutum* transformants expressing *CrBKT*. Figure S3: Western blot analysis of GFP-fused CrBKT expression constructs (P4, P5); Figure S4: Uncropped Western blot membranes for CrBKT protein detection; Table S1: Primers used in this study; Table S2: Detected metabolites in the wild type and transgenic *P. tricornutum*; Table S3: The comparison of metabolites between WT and O6, A3.

## Abbreviations

The following abbreviations are used in this manuscript:

BKT	β-carotene ketolase
CHY	β-carotene hydroxylase
LCYB	lycopene β-cyclase
WT	Wild Type
DCW	Dry Cell Weight
qPCR	Quantitative Polymerase Chain Reaction
RT-qPCR	Reverse Transcription Quantitative Polymerase Chain Reaction
TBS	TATA box binding protein
HA	Hemagglutinin (epitope tag)
GFP	Green Fluorescent Protein
ER	Endoplasmic Reticulum
SDS-PAGE	Sodium Dodecyl Sulfate–Polyacrylamide Gel Electrophoresis
PCA	Principal Component Analysis
ECL	Enhanced Chemiluminescence
FC	Fold Change
FDR	False Discovery Rate
BHT	Butylated Hydroxytoluene
APCI	Atmospheric Pressure Chemical Ionization

## References

[1] Ambati R.R., Moi P.S., Ravi S., Aswathanarayana R.G. (2014). Astaxanthin: Sources, Extraction, Stability, Biological Activities and Its Commercial Applications—A Review. Mar. Drugs.

[2] Youssef F.M., Ateyya H., Hanna Samy A.E., Elmokadem E.M. (2025). The Anti-Inflammatory and Antioxidant Effects of Astaxanthin as an Adjunctive Therapy in Community-Acquired Pneumonia: A Randomized Controlled Trial. Front. Pharmacol..

[3] Solovchenko A.E. (2015). Recent Breakthroughs in the Biology of Astaxanthin Accumulation by Microalgal Cell. Photosynth. Res..

[4] Do T.T., Ong B.N., Tran M.L.N., Nguyen D., Melkonian M., Tran H.D. (2019). Biomass and Astaxanthin Productivities of *Haematococcus pluvialis* in an Angled Twin-Layer Porous Substrate Photobioreactor: Effect of Inoculum Density and Storage Time. Biology.

[5] Rizzo A., Ross M.E., Norici A., Jesus B. (2022). A Two-Step Process for Improved Biomass Production and Non-Destructive Astaxanthin and Carotenoids Accumulation in *Haematococcus pluvialis*. Appl. Sci..

[6] Shah M.M.R., Liang Y., Cheng J.J., Daroch M. (2016). Astaxanthin-Producing Green Microalga *Haematococcus pluvialis*: From Single Cell to High Value Commercial Products. Front. Plant Sci..

[7] Debnath T., Bandyopadhyay T.K., Vanitha K., Bobby M.N., Nath Tiwari O., Bhunia B., Muthuraj M. (2024). Astaxanthin from Microalgae: A Review on Structure, Biosynthesis, Production Strategies and Application. Food Res. Int..

[8] Butler T., Kapoore R.V., Vaidyanathan S. (2020). *Phaeodactylum tricornutum*: A Diatom Cell Factory. Trends Biotechnol..

[9] Wang S., Hu Z. (2025). The Marine Diatom *Phaeodactylum tricornutum* as a Versatile Bioproduction Chassis: Current Progress, Challenges and Perspectives. Plant Commun..

[10] Yang R., Wei D. (2020). Improving Fucoxanthin Production in Mixotrophic Culture of Marine Diatom *Phaeodactylum tricornutum* by LED Light Shift and Nitrogen Supplementation. Front. Bioeng. Biotechnol..

[11] Elshobary M.E., Abo-Shanab W.A., Ende S.S.W., Alquraishi M., El-Shenody R.A. (2025). Optimizing *Phaeodactylum tricornutum* Cultivation: Integrated Strategies for Enhancing Biomass, Lipid, and Fucoxanthin Production. Biotechnol. Biofuels Bioprod..

[12] Tanaka K., Lan J.C.W., Kondo A., Hasunuma T. (2025). Metabolic Engineering and Cultivation Strategies for Efficient Production of Fucoxanthin and Related Carotenoids. Appl. Microbiol. Biotechnol..

[13] Kadono T., Kira N., Suzuki K., Iwata O., Ohama T., Okada S., Nishimura T., Akakabe M., Tsuda M., Adachi M. (2015). Effect of an Introduced Phytoene Synthase Gene Expression on Carotenoid Biosynthesis in the Marine Diatom *Phaeodactylum tricornutum*. Mar. Drugs.

[14] Manfellotto F., Stella G.R., Ferrante M.I., Falciatore A., Brunet C. (2020). Engineering the Unicellular Alga *Phaeodactylum tricornutum* for Enhancing Carotenoid Production. Antioxidants.

[15] Græsholt C., Brembu T., Volpe C., Bartosova Z., Serif M., Winge P., Nymark M. (2024). Zeaxanthin Epoxidase 3 Knockout Mutants of the Model Diatom *Phaeodactylum tricornutum* Enable Commercial Production of the Bioactive Carotenoid Diatoxanthin. Mar. Drugs.

[16] Athanasakoglou A., Kampranis S.C. (2019). Diatom Isoprenoids: Advances and Biotechnological Potential. Biotechnol. Adv..

[17] Zhong Y.J., Huang J.C., Liu J., Li Y., Jiang Y., Xu Z.F., Sandmann G., Chen F. (2011). Functional Characterization of Various Algal Carotenoid Ketolases Reveals That Ketolating Zeaxanthin Efficiently Is Essential for High Production of Astaxanthin in Transgenic Arabidopsis. J. Exp. Bot..

[18] He J., Li P., Huo H., Liu L., Tang T., He M., Huang J., Liu L. (2019). Heterologous Expression of HpBHY and CrBKT Increases Heat Tolerance in Physcomitrella Patens. Plant Divers..

[19] Bernaudat F., Frelet-Barrand A., Pochon N., Dementin S., Hivin P., Boutigny S., Rioux J.B., Salvi D., Seigneurin-Berny D., Richaud P. (2011). Heterologous Expression of Membrane Proteins: Choosing the Appropriate Host. PLoS ONE.

[20] Maier U.G., Zauner S., Hempel F. (2015). Protein Import into Complex Plastids: Cellular Organization of Higher Complexity. Eur. J. Cell Biol..

[21] Gruber A., Vugrinec S., Hempel F., Gould S.B., Maier U.G., Kroth P.G. (2007). Protein Targeting into Complex Diatom Plastids: Functional Characterisation of a Specific Targeting Motif. Plant Mol. Biol..

[22] Perozeni F., Cazzaniga S., Baier T., Zanoni F., Zoccatelli G., Lauersen K.J., Wobbe L., Ballottari M. (2020). Turning a Green Alga Red: Engineering Astaxanthin Biosynthesis by Intragenic Pseudogene Revival in *Chlamydomonas reinhardtii*. Plant Biotechnol. J..

[23] Diaz-Garza A.M., Lavoie-Marchand F., Merindol N., Diamond A., Desgagné-Penix I. (2024). What Is True for Plants May Not Be True for Phaeodactylum: The Case of *Vanilla planifolia* Vanillin Synthase (VpVAN) Targeted to Four Subcellular Compartments of the Diatom. bioRxiv.

[24] Zou L.G., Balamurugan S., Zhou T.B., Chen J.W., Li D.W., Yang W.D., Liu J.S., Li H.Y. (2019). Potentiation of Concurrent Expression of Lipogenic Genes by Novel Strong Promoters in the Oleaginous Microalga *Phaeodactylum tricornutum*. Biotechnol. Bioeng..

[25] Ye L., Zhu X., Wu T., Wang W., Zhao D., Bi C., Zhang X. (2018). Optimizing the Localization of Astaxanthin Enzymes for Improved Productivity. Biotechnol. Biofuels.

[26] Zhou Q., Huang D., Yang H., Hong Z., Wang C. (2024). Improvement of Carotenoids’ Production by Increasing the Activity of Beta-Carotene Ketolase with Different Strategies. Microorganisms.

[27] Bai C., Berman J., Farre G., Capell T., Sandmann G., Christou P., Zhu C. (2017). Reconstruction of the Astaxanthin Biosynthesis Pathway in Rice Endosperm Reveals a Metabolic Bottleneck at the Level of Endogenous β-Carotene Hydroxylase Activity. Transgenic Res..

[28] Dambek M., Eilers U., Breitenbach J., Steiger S., Büchel C., Sandmann G. (2012). Biosynthesis of Fucoxanthin and Diadinoxanthin and Function of Initial Pathway Genes in *Phaeodactylum tricornutum*. J. Exp. Bot..

[29] Bai Y., Cao T., Dautermann O., Buschbeck P., Cantrell M.B., Chen Y., Lein C.D., Shi X., Ware M.A., Yang F. (2022). Green Diatom Mutants Reveal an Intricate Biosynthetic Pathway of Fucoxanthin. Proc Natl Acad Sci. USA.

[30] Oslan S.N.H., Shoparwe N.F., Yusoff A.H., Rahim A.A., Chang C.S., Tan J.S., Oslan S.N., Arumugam K., Bin Ariff A., Sulaiman A.Z. (2021). A Review on *Haematococcus pluvialis* Bioprocess Optimization of Green and Red Stage Culture Conditions for the Production of Natural Astaxanthin. Biomolecules.

[31] Gherabli A., Grimi N., Lemaire J., Vorobiev E., Lebovka N. (2023). Extraction of Valuable Biomolecules from the Microalga *Haematococcus pluvialis* Assisted by Electrotechnologies. Molecules.

[32] Delgado-Ramallo J.F., Álvarez-Gil M., Casado-Bañares V., Suárez-Montes D., Sanjurjo-Muñíz C. (2024). *Phaeodactylum tricornutum* as a Stable Platform for Pilot Scale Production and Investigation of the Viability of Spirulina Fucoxanthin as a Commercial Lipolysis Active Novel Compound. Front. Mar. Sci..

[33] Fang H., Liu J., Ma R., Zou Y., Ho S.H., Chen J., Xie Y. (2023). Functional Characterization of Lycopene β- and ε-Cyclases from a Lutein-Enriched Green Microalga Chlorella Sorokiniana FZU60. Mar. Drugs.

[34] Cen S.Y., Li D.W., Huang X.L., Huang D., Balamurugan S., Liu W.J., Zheng J.W., Yang W.D., Li H.Y. (2022). Crucial Carotenogenic Genes Elevate Hyperaccumulation of Both Fucoxanthin and β-Carotene in *Phaeodactylum tricornutum*. Algal Res..

[35] Henke N.A., Heider S.A.E., Peters-Wendisch P., Wendisch V.F. (2016). Production of the Marine Carotenoid Astaxanthin by Metabolically Engineered Corynebacterium Glutamicum. Mar. Drugs.

[36] Wu Y., Yan P., Liu X., Wang Z., Tang Y.J., Chen T., Zhao X. (2019). Combinatorial Expression of Different β-Carotene Hydroxylases and Ketolases in Escherichia Coli for Increased Astaxanthin Production. J. Ind. Microbiol. Biotechnol..

[37] Menin B., Lami A., Musazzi S., Petrova A.A., Santabarbara S., Casazza A.P. (2019). A Comparison of Constitutive and Inducible Non-Endogenous Keto-Carotenoids Biosynthesis in *Synechocystis* sp. PCC 6803. Microorganisms.

[38] Vidhyavathi R., Venkatachalam L., Sarada R., Ravishankar G.A. (2008). Regulation of Carotenoid Biosynthetic Genes Expression and Carotenoid Accumulation in the Green Alga *Haematococcus pluvialis* under Nutrient Stress Conditions. J. Exp. Bot..

[39] Chen Q., Chen Y., Xu Q., Jin H., Hu Q., Han D. (2022). Effective Two-Stage Heterotrophic Cultivation of the Unicellular Green Microalga Chromochloris Zofingiensis Enabled Ultrahigh Biomass and Astaxanthin Production. Front. Bioeng. Biotechnol..

[40] Lyu X., Lyu Y., Yu H., Chen W.N., Ye L., Yang R. (2022). Biotechnological Advances for Improving Natural Pigment Production: A State-of-the-Art Review. Bioresour. Bioprocess..

[41] Giossi C.E., Kroth P.G., Lepetit B. (2025). Xanthophyll Cycling and Fucoxanthin Biosynthesis in the Model Diatom *Phaeodactylum tricornutum*: Recent Advances and New Gene Functions. Front. Photobiol..

[42] Cao T., Bai Y., Buschbeck P., Tan Q., Cantrell M.B., Chen Y., Jiang Y., Liu R.Z., Ries N.K., Shi X. (2023). An Unexpected Hydratase Synthesizes the Green Light-Absorbing Pigment Fucoxanthin. Plant Cell.

[43] Cerón-García M.C., Fernández-Sevilla J.M., Sánchez-Mirón A., García-Camacho F., Contreras-Gómez A., Molina-Grima E. (2013). Mixotrophic Growth of *Phaeodactylum tricornutum* on Fructose and Glycerol in Fed-Batch and Semi-Continuous Modes. Bioresour. Technol..

[44] Zaslavskaia L.A., Lippmeier J.C., Shih C., Ehrhardt D., Grossman A.R., Apt K.E. (2001). Trophic Conversion of an Obligate Photoautotrophic Organism Through Metabolic Engineering. Science.

[45] Guillard R.R.L., Smith W.L., Chanley M.H. (1975). Culture of Phytoplankton for Feeding Marine Invertebrates. Culture of Marine Invertebrate Animals.

[46] Zaslavskaia L.A., Casey Lippmeier J., Kroth P.G., Grossman A.R., Apt K.E. (2000). Transformation of the Diatom *Phaeodactylum tricornutum* (Bacillariophyceae) with a Variety of Selectable Marker and Reporter Genes. J. Phycol..

[47] Apt K.E., Kroth-Pancic Arthur R Grossman P.G. (1996). Stable Nuclear Transformation of the Diatom *Phaeodactylum tricornutum*. Mol. Gen. Genet..

[48] Sachse M., Sturm S., Gruber A., Kroth P.G. (2013). Identification and evaluation of endogenous reference genes for steady state transcript quantification by qPCR in the diatom *Phaeodactylum tricornutum* with constitutive expression independent from time and light. Endocytobiosis Cell Res..

[49] Geyer R., Peacock A.D., White D.C., Lytle C., Van Berkel G.J. (2004). Atmospheric Pressure Chemical Ionization and Atmospheric Pressure Photoionization for Simultaneous Mass Spectrometric Analysis of Microbial Respiratory Ubiquinones and Menaquinones. J. Mass Spectrom..

