# Supplementary material for: Installing a Ketocarotenoid Branch in Phaeodactylum tricornutum via Functional Activation of Chlamydomonas reinhardtii β-Carotene Ketolase
Source: Mar Drugs. 2025 Dec 8;23(12):470. doi: 10.3390/md23120470 (PMC12735154; doi:10.3390/md23120470)
Supplement: Supplementary file 1 [file marinedrugs-23-00470-s001.zip › marinedrugs-4027008-supplementary.pdf]

# Supplementary Materials

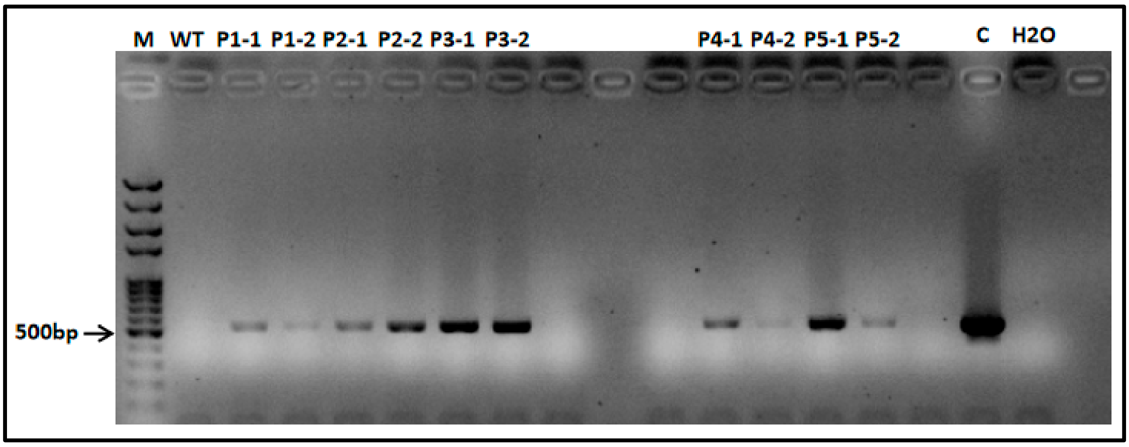


**Figure S1**. PCR-based confirmation of CrBKT gene integration in transgenic *P. tricornutum* lines. Genomic DNA was extracted from wild-type (WT) and representative transformants (P1–P5 series). PCR was performed using CrBKT-specific primers to detect integration of the transgene. A single band of expected size (~500 bp) was observed in transformants but absent in WT and negative control ($H_2O$). Plasmid DNA (C) was used as a positive control. M: DNA ladder.

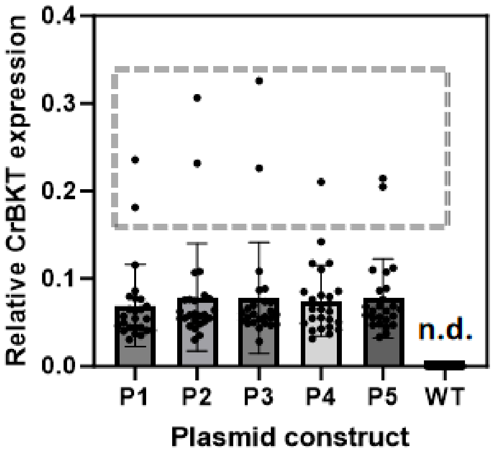


**Figure S2.** qRT-PCR–based screening of *P. tricornutum* transformants expressing CrBKT. Independent transformants generated with plasmid constructs P1–P5 were analyzed by qRT-PCR using the TBS gene as an internal reference. Relative CrBKT transcript levels were calculated as $2^{-\Delta Ct}$ ($\Delta$Ct = Ct_CrBKT − Ct_TBS). Bars represent the mean ± SD of all transformants obtained with each construct, and dots indicate individual transformants, CrBKT transcripts were not detected in the wild-type (WT) strain (n.d., Ct > 40), which is therefore shown at zero for visualization. The top two high-expressing clones for each construct (boxed) were selected for subsequent Western blot and carotenoid analyses.

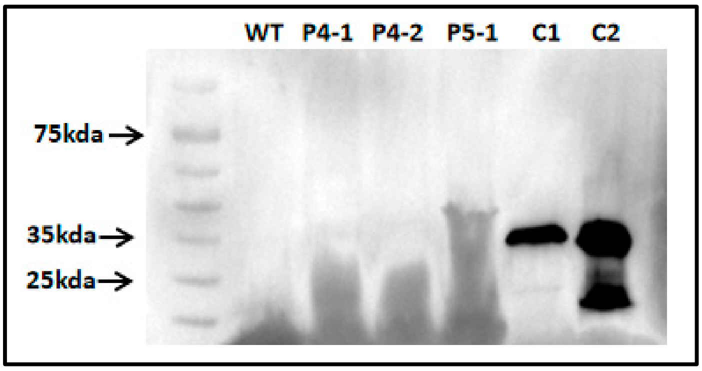


**Figure S3.** Western blot analysis of GFP-fused CrBKT expression constructs (P4, P5). Total protein extracts from wild-type (WT), representative transformants (P4-1, P4-2, P5-1), and GFP-only positive controls (C1, C2) were probed with an anti-GFP-HRP monoclonal antibody. No detectable GFP-fused CrBKT protein bands were observed in the engineered P4 and P5 lines, while strong bands were detected in the GFP-only control lines (C1, C2), validating antibody performance. The absence of fusion protein bands suggests instability or degradation of the CrBKT-GFP fusion proteins.

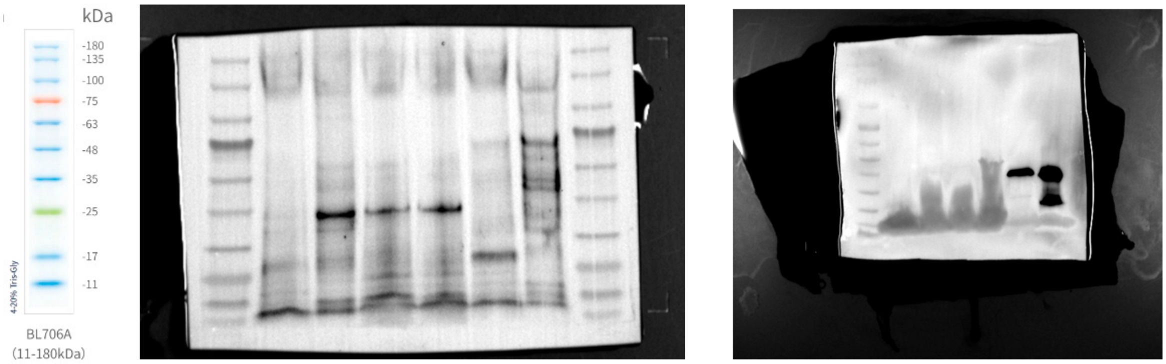


**Figure S4.** Uncropped Western blot membranes for CrBKT protein detection. Left: Full blot corresponding to anti-HA detection of CrBKT in wild-type (WT) and plastid-targeted transformants (P2, P3, P1-1, P1-2). Right: Full blot corresponding to anti-GFP detection of CrBKT-GFP fusion constructs (P4, P5) and positive GFP-only controls (C1, C2). Ladder bands are shown on both sides of each blot for reference. These full-membrane scans validate the cropped images shown in the main and supplementary figures and confirm specificity of antibody-based detection.

**Table S1**. Primers used in this study.

| **Primer name** | **Primer sequence（5'→3'）** | Used for | Produce size(bp) |
|---|---|---|---|
| CrBKT-F | TGTGGTTTGTCAAGTTGCCGT | PCR identification of CrBKTgene | 571 |
| CrBKT-R | GTAAGAAAGGACTGGAGACGC | | |
| TBP-F | ATCGATTTGTCAATCCACGAG | Internal control for RT-PCR | 213 |
| TBP-R | ATACAGATTCTGTGTCCACGG | | |
| qCrBKT-F | GACCCAGATTTCCACCGAGG | qPCR identification of CrBKTgene | 151 |
| qCrBKT-R | CAACAACTGGTTGGCGAGTG | | |

**Table S2.** Detected metabolites in the wild type and transgenic Phaeodactylum tricornutum.Unit: μg/g.

| | Metabolite | WT-R1 | WT-R2 | WT-R3 | O6-R1 | O6-R2 | O6-R3 | A3-R1 | A3-R2 | A3-R3 | P1-1 | P1-2 | P1-3 |
|---|---|---|---|---|---|---|---|---|---|---|---|---|---|
| 1 | Fucoxanthin | 3910.82426 | 3602.35946 | 3545.20439 | 1508.16958 | 1467.38218 | 1432.25843 | 1374.21111 | 1332.26136 | 1383.89724 | 3573.32353 | 3403.41743 | 3519.43303 |
| 2 | Antheraxanthin | 23.4769829 | 18.1670964 | 18.6180123 | 19.9978803 | 18.0433358 | 15.032526 | 23.9157068 | 22.6268109 | 23.498738 | 19.2109874 | 27.6660264 | 22.1531886 |
| 3 | β-Carotene | 416.158631 | 331.315111 | 326.416709 | 145.506359 | 130.384389 | 117.302815 | 187.578179 | 176.531752 | 199.344351 | 364.57563 | 384.733372 | 345.380888 |
| 4 | β-Cryptoxanthin | 10.2278383 | 9.46740588 | 8.79121104 | 4.89239401 | 4.74053756 | 4.15191479 | 5.51948895 | 5.83630681 | 6.05796274 | 8.47856092 | 8.8977638 | 8.69133166 |
| 5 | Violaxanthin | 28.609409 | 21.9157813 | 20.8785897 | 36.6676309 | 35.1801178 | 31.7615014 | 28.5165484 | 26.7523913 | 25.5283053 | 18.47856092 | 20.8977638 | 22.3487594 |
| 6 | Zeaxanthin | 14.8845257 | 12.4616813 | 12.5512519 | 5.13785536 | 4.79351988 | 4.0528912 | 6.78175826 | 6.2747569 | 6.99969952 | 12.31228992 | 12.3682913 | 11.1534697 |
| 7 | γ-Carotene | 0 | 0 | 0 | 0.558396509 | 0.56582106 | 0.447561501 | 0.708686879 | 0.571369319 | 0.566646635 | 0 | 0 | 0 |
| 8 | Lycopene | 0 | 0 | 0 | 1.17201621 | 1.15985272 | 1.12270226 | 1.1282032 | 1.16333796 | 1.03195313 | 0 | 0 | 0 |
| 9 | Echinenone | 0 | 0 | 0 | 11.2811596 | 11.0959499 | 10.1157367 | 9.48848104 | 9.13333003 | 8.13786058 | 0.023264748 | 0.01923449 | 0.016175491 |
| 10 | Adonirubin | 0 | 0 | 0 | 369.095012 | 357.401325 | 333.546285 | 403.177854 | 371.279024 | 401.09976 | 0 | 0 | 0 |
| 11 | Canthaxanthin | 0 | 0 | 0 | 45.686783 | 44.482327 | 44.3681207 | 39.8392821 | 38.3382616 | 38.9035457 | 0.029828698 | 0.022876634 | 0.029809404 |
| 12 | Astaxanthin | 0 | 0 | 0 | 14.412419 | 13.4529455 | 12.2762617 | 16.1284831 | 15.7267513 | 16.2794772 | 0 | 0 | 0 |

**Table S3.** The comparison of metabolites between WT and O6,A3

| O6 vs WT | Metabolite | P-value | FDR | FC | Log2FC | Type | A3 vs WT | Metabolite | P-value | FDR | FC | Log2FC | Type |
|---|---|---|---|---|---|---|---|---|---|---|---|---|---|
| 1 | Fucoxanthin | 0.001929365 | 0.002968253 | 0.398594275 | -1.327007106 | down | 1 | Fucoxanthin | 0.002022309 | 0.005399766 | 0.36988842 | -1.434837958 | down |
| 2 | Antheraxanthin | 0.344645376 | 0.344645376 | 0.880715234 | -0.183252474 | insig | 2 | Antheraxanthin | 0.190541181 | 0.20006824 | 1.16227721 | 0.216954201 | insig |
| 3 | β-Carotene | 0.011395262 | 0.012661402 | 0.366139361 | -1.449535221 | down | 3 | β-Carotene | 0.023512768 | 0.025987796 | 0.524685066 | -0.930476368 | up |
| 4 | β-Cryptoxanthin | 0.001680747 | 0.002801245 | 0.4839088 | -1.04719292 | down | 4 | β-Cryptoxanthin | 0.006447685 | 0.010622985 | 0.611299594 | -0.710048487 | down |
| 5 | Violaxanthin | 0.027437454 | 0.02888153 | 1.451033126 | 0.537080455 | insig | 5 | Violaxanthin | 0.325838618 | 0.325838618 | 1.13155417 | 0.178305651 | insig |
| 6 | Zeaxanthin | 0.003531833 | 0.004414791 | 0.350505191 | -1.512492285 | down | 6 | Zeaxanthin | 0.009987322 | 0.01233728 | 0.502694037 | -0.992247519 | up |
| 7 | γ-Carotene | 0.005285656 | 0.006218419 | Inf | Inf | up | 7 | γ-Carotene | 0.005677134 | 0.010622985 | Inf | Inf | up |
| 8 | Lycopene | 1.66E-04 | 0.00116314 | Inf | Inf | up | 8 | Lycopene | 0.001254266 | 0.004389932 | Inf | Inf | up |
| 9 | Echinenone | 0.001116946 | 0.002030811 | 516.2447286 | 9.011911335 | up | 9 | Echinenone | 0.002057054 | 0.005399766 | 425.1563358 | 8.731849627 | up |
| 10 | Adonirubin | 8.75E-04 | 0.001750422 | Inf | Inf | up | 10 | Adonirubin | 6.91E-04 | 0.003425151 | Inf | Inf | up |
| 11 | Canthaxanthin | 8.85E-05 | 0.00116314 | Inf | Inf | up | 11 | Canthaxanthin | 1.26E-04 | 0.001320376 | Inf | Inf | up |
| 12 | Astaxanthin | 0.002124449 | 0.003034927 | Inf | Inf | up | 12 | Astaxanthin | 1.06E-04 | 0.001320376 | Inf | Inf | up |
